# The synapse in traumatic brain injury

**DOI:** 10.1093/brain/awaa321

**Published:** 2020-11-13

**Authors:** Aimun A B Jamjoom, Jonathan Rhodes, Peter J D Andrews, Seth G N Grant

**Affiliations:** 1 Centre for Clinical Brain Sciences, Chancellor's Building, Edinburgh BioQuarter, University of Edinburgh, Edinburgh EH16 4SB, UK; 2 Anaesthesia, Critical Care and Pain Medicine, University of Edinburgh, Edinburgh EH16 4SA, UK; 3 Simons Initiative for the Developing Brain (SIDB), Centre for Discovery Brain Sciences, University of Edinburgh, Hugh Robson Building, George Square, Edinburgh EH8 9XD, UK

**Keywords:** synaptopathy, synaptome, astrocyte, inflammation, microglia

## Abstract

Traumatic brain injury (TBI) is a leading cause of death and disability worldwide and is a risk factor for dementia later in life. Research into the pathophysiology of TBI has focused on the impact of injury on the neuron. However, recent advances have shown that TBI has a major impact on synapse structure and function through a combination of the immediate mechanical insult and the ensuing secondary injury processes, leading to synapse loss. In this review, we highlight the role of the synapse in TBI pathophysiology with a focus on the confluence of multiple secondary injury processes including excitotoxicity, inflammation and oxidative stress. The primary insult triggers a cascade of events in each of these secondary processes and we discuss the complex interplay that occurs at the synapse. We also examine how the synapse is impacted by traumatic axonal injury and the role it may play in the spread of tau after TBI. We propose that astrocytes play a crucial role by mediating both synapse loss and recovery. Finally, we highlight recent developments in the field including synapse molecular imaging, fluid biomarkers and therapeutics. In particular, we discuss advances in our understanding of synapse diversity and suggest that the new technology of synaptome mapping may prove useful in identifying synapses that are vulnerable or resistant to TBI.

## Teaser

Traumatic brain injury is a leading cause of death and disability and is a risk factor for dementia later in life. Jamjoom *et al.* review the role of the synapse in TBI pathophysiology with a focus on the confluence of secondary injury processes including axonal injury, excitotoxicity, inflammation and oxidative stress.

## Introduction

Traumatic brain injury (TBI) is a temporary or permanent impairment of brain function due to an external mechanical force to the head. It is a leading cause of death and disability worldwide, with a rising global incidence due to increased use of motor vehicles in low and middle income countries (Maas *et al.*, 2008). TBI leads to immediate functional impairment and there is growing epidemiological evidence that TBI is major risk factor for dementia later in life (Mortimer *et al.*, 1985; Graves *et al.*, 1990; Fann *et al.*, 2018; Gorgoraptis *et al.*, 2019). There is also evidence that TBI may be a risk factor for Parkinson’s disease and stroke (Chen *et al.*, 2011; Gardner *et al.*, 2018). Although most research into the mechanisms of TBI has focused on neuronal damage, recent advances have shown that synapses and their intrinsic molecular pathways play a major role in a diverse range of brain diseases, raising the possibility that these may be involved with TBI. Studies of the synapse proteome and genome sequencing of patients with developmental, psychiatric and neurological disorders reveal that over 130 brain disorders arise from mutations disrupting synapse proteins (Husi *et al.*, 2000; Grant *et al.*, 2005; Pocklington *et al.*, 2006; Fernández *et al.*, 2009, 2017; Feyder *et al.*, 2010; Bayés *et al.*, 2011; Fromer *et al.*, 2014; Purcell *et al.*, 2014; Reig-Viader *et al.*, 2018; MacDonald *et al.*, 2020). The remarkable burden and diversity of synaptic diseases arising from these mutations highlights the importance of maintaining appropriate levels of synapse proteins for normal brain function. The postsynaptic proteome of excitatory synapses comprises >1000 proteins that are highly conserved between vertebrate species (Bayés *et al.*, 2011, 2012, 2017). These proteins are of many different structural and functional classes, including neurotransmitter receptors, ion channels, scaffolding, signalling and cytoskeletal proteins (Husi *et al.*, 2000; Peng *et al.*, 2004; Collins *et al.*, 2006; Emes *et al.*, 2008; Bayés *et al.*, 2011, 2012, 2017; Distler *et al.*, 2014, Roy *et al.*, 2018*a*, *b*).

Alongside evidence of the role of mutations in genes encoding synapse proteins as the cause of a range of intrinsic brain disorders, synapses also appear to be part of the early pathophysiology of inflammatory and degenerative disorders such as multiple sclerosis and Alzheimer’s disease (Wegner *et al.*, 2006; Hong *et al.*, 2016). In Alzheimer’s disease, synapse loss correlates with cognitive decline and is present in the prodromal stage of the disease (Scheff *et al.*, 2006; Sultana *et al.*, 2010). This is important as there is growing evidence that TBI, particularly repetitive injuries such as those experienced by boxers and American football players, leads to a neurodegenerative condition termed chronic traumatic encephalopathy (CTE) (Stern *et al.*, 2013). CTE pathology is characterized by hyperphosphorylated tau protein as neurofibrillary tangles, particularly within sulcal depths (McKee *et al.*, 2015). In Alzheimer’s disease, synapses appear to play a role in disease progression through the trans-synaptic spread of pathological tau (Spires-Jones and Hyman, 2014). This raises the question of whether synapses play a role in the progression of neurodegeneration following TBI.

**Figure 1:**
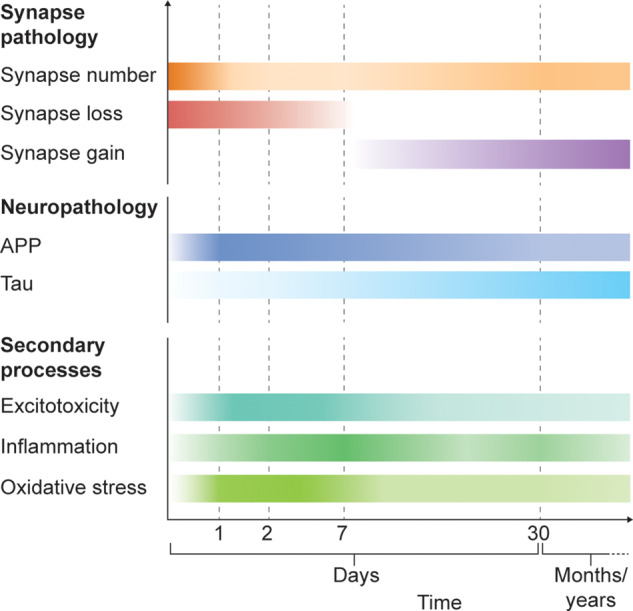
**Summary of pathological changes following traumatic brain injury.** After TBI, synapse density is reduced for up to 7 days, followed by a recovery which plateaus around 30 days (Scheff *et al.*, 2005). Abnormal accumulation of APP peaks 1 day after TBI but can persist for months to years after injury (Johnson *et al.*, 2013). Tau spreads through the brain after TBI and neurofibrillary tangles are a hallmark of chronic traumatic encephalopathy (Edwards *et al.*, 2020). There is a rapid rise in extracellular glutamate after TBI, and levels may remain abnormally elevated up to a week from the injury (Vespa *et al.*, 1998; Hong *et al.*, 2001). Microglia show bimodal peaks after TBI at 7 days and then 30 days (Simon *et al.*, 2017). Markers of oxidative stress appear rapidly after injury and may persist up to 6 months after TBI (Licastro *et al.*, 2016).

In this review, we examine the role of the synapse in TBI pathophysiology and draw attention to the confluence of secondary injury processes that target synapses, the potential mechanisms of synaptic loss and recovery after a traumatic insult, and the role that neuroinflammatory cells play in this process (Fig. 1). Coupled to this, we look at how the synapse may contribute to disease through the trans-synaptic spread of tau. Finally, we explore the direction in which the burgeoning field of traumatic synaptopathy is heading.

## Traumatic injury leads to rapid changes in synapse proteins and synapse loss

Early clues as to the effect of neuronal death on synapses came from electron microscopy (EM) ultrastructural studies that monitored synapse changes after denervation (Anderson *et al.*, 1986; Marrone *et al.*, 2004). Neuron injury through intraventricular injections of kainic acid or electroablation demonstrated a significant reduction in synaptic density 3–4 days post lesion (Anderson *et al.*, 1986; Marrone *et al.*, 2004). However, chemical lesions and electroablation may use different pathways and mechanisms to TBI induced by mechanical force. To overcome this, several preclinical studies have used biochemical and microscopy methods to quantify synapse loss in animal models of TBI (Table 1). In a controlled cortical impact (CCI) model, Scheff *et al.* (2005) used EM to examine synaptic (presynaptic terminal and postsynaptic density) changes in the CA1 stratum radiatum of the hippocampal formation and found the injury group had 44% synaptic density compared with sham injury mice at 2 days post injury. Gao *et al.* (2011) examined presynaptic punctum number measured with synaptophysin and dendritic density in the dentate gyrus using light microscopy and immunohistochemistry and found the injury group had 69% synapse density compared with sham after a CCI. The injury induced limited neuronal death but significant reduction in dendritic density, highlighting that substantial synapse loss can occur even without overt neuronal death. Dendritic spine loss without neuronal death has been found in other TBI preclinical studies (Posmantur *et al.*, 1996; Huh *et al.*, 2003). Together, these studies support the view that TBI induces synapse loss within the first 48 h post injury within the hippocampus ipsilateral to the injury.

**Table 1:** Summary of animal studies examining synaptic changes after TBI

Injury model			Species	Time after injury	Assay	Marker	Synapse pathology	Reference
**Controlled cortical impact**								
Injury severity								
Diameter, mm	Depth, mm	Velocity, m/s						
3	1	3.0	Mouse	1–7 days	IHC	Synaptophysin	Molecular layer of the hippocampal dentate gyrus; 30% loss in presynaptic puncta density at 3 days	Gao *et al.*, 2011
3	1	4.5	Mouse	1–7 days	WB	PSD95	Hippocampus; postsynaptic PSD95 reduced at 7 days	Wakade *et al.*, 2010
3	1	6.0	Mouse	1–7 days	IHC	VGlut1	Hippocampus CA1 stratum radiatum; 27% loss in presynaptic puncta density 3 days	Nikolakopoulou *et al*., 2016
5	2	3.5	Rat	2–60	EM	N/A	Hippocampus CA1 stratum radiatum; 66% synapses lost at 2 days; 14% synapses lost at 30 days	Scheff *et al.*, 2005
5	2	3.5	Rat	3 h–4 days	WB	Synapsin; PSD95; SAP97; GAP43	Neocortex; presynaptic synapsin reduced from 1 to 4 days; postsynaptic PSD95 reduced from 1 to 4 days; SAP97 reduced at 4 days; GAP43 no change	Ansari *et al.*, 2008*a*
5	2	3.5	Rat	1 h–4 days	WB	Synapsin; PSD95 SAP97; GAP43	Hippocampus; postsynaptic PSD95 reduced from 2 days; presynaptic synapsin reduced from 2 days; SAP97 reduced at 4 days; GAP43 no change	Ansari *et al.*, 2008*b*
**Lateral fluid percussion injury**								
Injury severity								
Low (1.9 ± 0.2 atm) Moderate (3.3 ± 0.3 atm) High (6.0 ± 0.5 atm)			Rat	2–30 days	WB IHC	Synaptophysin	Neocortex; increased presynaptic synaptophysin IHC with increasing severity of injury; no difference in synaptophysin WB between sham and moderate injury from 2 to 30 days	Shojo and Kibayashi, 2006
**Rotatory head model**								
Undefined			Rat	1–30 days	EM	N/A	Sensorimotor cortex; 14% synapses lost at 1 day; 36% synapses lost at 7 days; 18% synapses lost at 30 days	Semchenko *et al.*, 2006
**Blast injury**								
15.8 psi	5.9 ms	1.4 Mach	Mouse	1–30 days	WB	PSD95	Cerebellum; PSD95 reduced at 30 days	Meabon *et al*., 2016

IHC = immunohistochemistry; N/A = not available/applicable; WB = western blot.

A limitation of many of these studies is that they only examined single subregions within the brain and therefore provided limited conclusions about the potential differential effects of TBI on other brain regions. Biochemical approaches provide a more global picture of changes in synaptic protein levels; however, they are limited by the loss of spatial information as part of the tissue homogenization process. Using western blotting in a CCI model, Wakade *et al.* (2010) found that 7 days after injury there was a 47% reduction in postsynaptic density protein 95 (PSD95, a postsynaptic protein) expression in the hippocampus. A similar study assessed several synaptic proteins (PSD95, synapsin 1, SAP97 and GAP43) in the hippocampus at multiple time points post CCI using western blotting (Ansari *et al.*, 2008*b*) and found significant reductions in PSD95 and synapsin 1 (a presynaptic protein) at 48 h, which persisted until 96 h post injury. Yu *et al.* (2015) corroborated these findings using a systems biology approach involving gene expression data from four different TBI animal models and found a suppressed network of synaptic proteins centred around PSD95. Although limited, there have been observations of synaptopathy in human cases of TBI. Castejòn *et al.* (1995) undertook a small case series examining human cortical tissue samples after TBI. Using EM, the authors found evidence of swollen and shrunken presynaptic terminals and separation from the postsynaptic density. The authors also described dendritic swelling and evidence of synaptic phagocytosis by astroglial cells. These studies provide useful insights into the impact of TBI on the synapse. However, any conclusions are limited by the preponderance of murine TBI models and the use of post-mortem tissue, which only provides a snapshot of the state of the synapse after injury. Coupled to this, the imaging and biochemical approaches used provide only limited spatial information.

Taking these limitations into account, these data point towards a vulnerability of synapses to traumatic insult, with evidence of synapse loss from 2 to 7 days post injury across differing TBI preclinical models and quantification techniques (Fig. 1). Together, these studies converge on the view that TBI results in the loss of synapses as well as a reduction in the expression of synapse proteins and their mRNAs.

## Relationship between traumatic axonal injury and synaptopathy

It is important to consider the mechanical damage caused to the axon and how this impacts on synapse number and function. TBI induces dynamic deformation of long white matter tracts resulting in disruption of the axons (Johnson *et al.*, 2013). This can either lead to primary axotomy or partial damage leading to progressive axonal dysfunction and delayed axonal degeneration (Hill *et al.*, 2016). Historically, traumatic axonal injury was determined using histological approaches such as haematoxylin and eosin or silver staining (Smith *et al.*, 2013*a*). However, the introduction of immunohistochemistry allowed for more specific labelling of proteins transported in axons. For example, amyloid precursor protein (APP) moves along the axon by anterograde transport (Smith *et al.*, 2013*a*). Mechanical perturbation of the axon leads to pooling of APP, and accumulation of this transported material leads to either varicosities along the length of the axon or, in the event of complete disconnection, an axonal bulb (Johnson *et al.*, 2013). Using APP as a marker, axonal injury appears to peak at 24 h and subsequently declines, although evidence of pathology can persist months after TBI (Fig. 1) (Johnson *et al.*, 2013). Canty and colleagues used two-photon microscopy and laser microsurgery to examine the effect of axotomy on presynaptic bouton dynamics *in vivo* at a single-axon level (Canty *et al.*, 2013*b*). The authors observed disappearance of the axon distal to the injury but also, very importantly, rapid reductions in bouton density along the injured proximal axon within 6 h. They also found that the degree of synaptic loss was dependent on the cell type and the prelesion axon structural dynamics. This suggests that damage to the axon interferes with the synapses located proximal and distal to the lesion site.

Axons within a single specimen can be found in differing stages of degeneration and disconnection, highlighting that TBI-induced damage is an evolving pathology (Johnson *et al.*, 2013). The pathophysiology of this process is believed to be related to dysregulation of Na^+^ channels, leading to a Ca^2+^ influx. The accumulation in Ca^2+^, which is due to both influx and the release from intracellular stores (Wolf *et al.*, 2001; Staal *et al.*, 2010), activates proteases, such as calpain, which proteolyse voltage-gated Na^+^ channels (von Reyn *et al.*, 2009). This process leads to loss of microtubules and energy failure secondary to mitochondrial damage and ultimately impairs axonal transport (Wolf *et al.*, 2001, Smith *et al.*, 2013*a*). In severe cases, axotomy and Wallerian degeneration occur within the distal axon.

Insight into the relationship of axonal damage and synapse loss has been aided by the Wallerian degeneration slow (Wld^s^) mutation, which is known to confer protection to axonal degeneration (Mack *et al.*, 2001) in a variety of degeneration-inducing models (Wang *et al.*, 2001; Gillingwater *et al.*, 2004; Sajadi *et al.*, 2004). In the context of trauma, a study using a cortical lesion model found evidence of degenerating presynaptic terminals in the striatum at Day 2 post lesion in wild-type mice, which peaked at Day 3 and had resolved by Day 10, but was delayed and less pronounced in Wld^s^ mice (Gillingwater *et al.*, 2006). The *Wld^s^* gene encodes the nicotinamide adenine dinucleotide (NAD^+^) biosynthetic enzyme nicotinamide mononucleotide adenylyltransferase 1 (NMNAT1) fused to an 18-amino acid sequence from the N-terminus of Ube4b (ubiquitylation enzyme) (Mack *et al.*, 2001). The Wld^s^ protein localizes to the neuron’s nucleus, where it potentially regulates both axonal and dendritic proteins (Mack *et al.*, 2001). Proteomic analysis demonstrated modified expression of 16 synapse proteins, suggesting that the Wld^s^ neuroprotective phenotype may be related to altered mitochondrial activity and changes to both the ubiquitin-proteasome and NAD-associated pathways (Wishart *et al.*, 2012). Thus, the protective effect of the *Wld^s^* gene on synapse loss may involve a postsynaptic mechanism.

A second major molecule of interest in axonal degeneration is sterile alpha and TIR motif-containing protein 1 (SARM1) (Osterloh *et al.*, 2012). SARM1 has been implicated in NAD^+^ metabolism, with evidence of suppressed NAD^+^ loss and axonal degeneration when SARM1 is knocked down (Gerdts *et al.*, 2015). A study examining the impact of SARM1 in TBI found evidence of attenuated traumatic injury and improved functional outcome in SARM1 knockout mice after CCI (Henninger *et al.*, 2016). Genetic ablation of SARM1 prevented loss of corticospinal axons after an acceleration injury (Ziogas and Koliatsos, 2018). Collectively, these data suggest a mechanistic role of both SARM1 and NMNAT1 in mediating traumatic axonal degeneration. Less is known about the impact of these molecules on traumatic synaptopathy. Axonal protection appears to minimise synaptic loss; however, the exact relationship between SARM1 and NMNAT1 has yet to be fully elucidated in the context of TBI.

## The synapse and secondary injury processes

Synaptopathy in TBI is likely to be a complex, dynamic and multifaceted process. The mechanical injury (primary insult) causes instantaneous tissue damage, which is followed by multiple secondary injury processes (Prins *et al.*, 2013). These begin within seconds to minutes after the primary injury and can last for months to years. In the following section, we discuss excitotoxicity, oxidative stress and inflammation and explore the role of the synapse in these processes.

### Glutamate excitotoxicity

**Figure 2:**
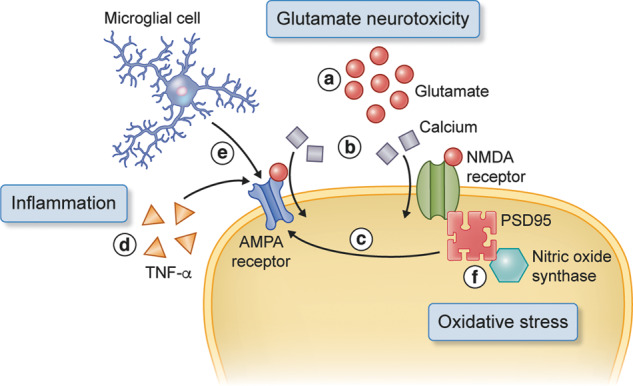
**TBI secondary injury and the postsynaptic density.** There is a surge of glutamate following TBI (a) (Vespa *et al.*, 1998; Hong *et al.*, 2001), which leads to influx of Ca^2+^ into the cell at the NMDA and AMPA receptors (b) (Nilsson *et al.*, 1996). PSD95, which is bound to PKCα, promotes GluR2-deficient AMPA receptors and exacerbates TBI excitotoxicity (c) (Spaethling *et al.*, 2008). The release of TNFα also acts upon the AMPA receptors and reduces expression of GluR2, which worsens excitotoxicity (d) (Beattie *et al.*, 2002; Stellwagen *et al.*, 2005). Triggered by CR3, microglia lead to long-term depression by activation of NADPH oxidase and GluR2-mediated AMPA receptor internalization (e) (Zhang *et al.*, 2014). PSD95 is bound to neuronal nitric oxide synthase which plays a role in promoting oxidative stress (f) (Brenman *et al.*, 1996).

Glutamate is the major neurotransmitter in the brain and is released from all excitatory synapses. Traumatic injury leads to an increase in extracellular glutamate levels, which appears within 24 h after injury and has been found to be abnormally elevated for up to a week in TBI patient CSF (Vespa *et al.*, 1998; Hong *et al.*, 2001) (Fig. 1). Glutamate acts upon two main neurotransmitter receptors on the postsynaptic terminal: NMDA receptors and AMPA receptors. These are ligand-gated ionotropic receptors, which play an important role in synaptic transmission and plasticity (Voglis and Tavernarakis, 2006; Paoletti *et al.*, 2013). AMPA receptors are principally involved in gating Na^+^ influx that depolarizes the postsynaptic neuron, whereas NMDA receptors gate the influx of Ca^2+^ crucial for signal transduction (Fig. 2). In TBI, elevated glutamate levels lead to abnormal intracellular Ca^2+^ levels and contribute to neuronal death (Nilsson *et al.*, 1996).

AMPA receptors consist of four subunits (GLuR1–4) and several studies have shown that TBI-related glutamate increases can lead to changes in AMPA receptors that perpetuate glutamate toxicity by exacerbating Ca^2+^ overload (Spaethling *et al.*, 2008; Bell *et al.*, 2009). The release of intracellular Ca^2+^ leads to the expression of AMPA receptors that lack the GluR2 subunit, making the receptor highly permeable to Ca^2+^ (Spaethling *et al.*, 2008). This shift in AMPA receptor subtype is an important factor in exacerbating excitotoxic neuronal death within an hour after injury. The NMDA receptor binds to PSD95, which in turn interacts with PKCα (Husi *et al.*, 2000; Frank *et al.*, 2016)—the protein kinase that phosphorylates the GluR2 subunit of the AMPA receptor—which induces GluR2-deficient AMPA receptors and exacerbates TBI excitotoxicity (Fig. 2).

### Oxidative stress

Oxidative stress arises from an overproduction of reactive oxygen species or dysfunction of the antioxidant system (Chen *et al.*, 2012). There is a range of reactive oxygen species, including hydroxyl, superoxide, nitric oxide and lipid peroxyl. The abundance of reactive oxygen species leads to cellular dysfunction through lipid peroxidation and DNA damage and has been implicated in a range of neurodegenerative diseases (Gandhi and Abramov, 2012). Evidence of oxidative stress following TBI has been found in both preclinical and human studies (Hall *et al.*, 1993; Cernak *et al.*, 2000; Tyurin *et al.*, 2000; Toczyłowska *et al.*, 2006; Wang *et al.*, 2016*a*) and has been found to persist up to 6 months after TBI (Licastro *et al.*, 2016) (Fig. 1). The synapse plays an important role in the convergence of glutamate toxicity and oxidative stress. Activation of the NMDA receptor and the influx of Ca^2+^ leads to the generation of reactive oxygen and nitrogen species (Szydlowska and Tymianski, 2010). In addition to the central role of PSD95 in coupling PKCα to NMDA receptors, it also interacts with neuronal nitric oxide synthase (nNOS), which generates nitric oxide (Brenman *et al.*, 1996) (Fig. 2), a free radical that plays a role in both inflammation and oxidative stress (Kobayashi, 2010; Cornelius *et al.*, 2013). Nitric oxide and peroxide form the reactive nitrogen species peroxynitrite, which is highly reactive and can nitrate tyrosine residues of proteins (Gandhi and Abramov, 2012). The physical coupling of nNOS to the NMDA receptor is key in oxidative damage, and disrupting PSD95 reduces cell death in an *in vitro* model of TBI (Arundine *et al.*, 2004; Bach *et al.*, 2019). Interestingly, a study using a systems biology approach to analyse gene expression data from multiple TBI animal models highlighted nNOS and PSD95 as potential protein markers of TBI (Yu *et al.*, 2015).

Two studies have also explored the relationship between oxidative stress and synaptic proteins in the cortex and hippocampus in a preclinical model of TBI (Ansari *et al.*, 2008*a*, *b*). A temporal analysis of post-mortem tissue showed evidence of oxidative stress with increased protein carbonyl, 3-nitrotyrosine and acrolein. These markers of oxidative stress preceded a reduction of PSD95 expression at 24 and 48 h post injury within the cortex and hippocampus, respectively. Although these findings do not support a causative relationship, this association suggests that the overwhelming of the antioxidant system after TBI may play a role in post-injury synaptopathy and warrants closer investigation.

There have been attempts to target oxidative stress as a therapeutic avenue in TBI. Magnesium has been investigated as it has a role as both a non-competitive NMDA receptor antagonist and has been shown to protect neurons against oxidative stress (Ruppersberg *et al.*, 1994; Regan *et al.*, 2002). A study of 31 TBI patients demonstrated a reduction in serum magnesium levels that negatively correlated with markers of oxidative stress (Cernak *et al.*, 2000). Coupled to this, preclinical studies showed that magnesium supplementation could ameliorate neuronal injury after TBI (Bareyre *et al.*, 2000; Saatman *et al.*, 2001). This led to a randomized clinical trial examining the efficacy of a magnesium sulphate infusion following TBI (Temkin *et al.*, 2007). The trial found that magnesium had no positive impact on clinical outcome following TBI and that the treatment group had an increase in medical complications. These findings have ruled out magnesium for the treatment of TBI.

### Inflammation

Inflammation is a well-described secondary injury process following TBI (Simon *et al.*, 2017). There is growing evidence that the inflammatory process acts upon the synapse to mediate neuronal damage. Tumour necrosis factor alpha (TNFα) is a proinflammatory cytokine that infiltrates the injury site and reduces expression of AMPA GluR2 subunits (Beattie *et al.*, 2002; Stellwagen *et al.*, 2005) (Fig. 2). As described above, these changes in AMPA structure can lead to Ca^2+^ overload and increase cell death. More recently, Zhang *et al.* (2014) found in an *in vitro* model of ischaemia and inflammation that activation of microglial complement receptor 3 (CR3) triggers long-term depression of synaptic transmission by activation of NADPH oxidase and internalization of GluR2-containing AMPA receptors (Zhang *et al.*, 2014) (Fig. 2). Long-term depression is thought to underlie cognitive functions (Bear and Abraham, 1996) and thus inflammatory processes may contribute to post-traumatic cognitive impairments.

A particularly important question is the role of glial cells in synaptopathy. Several studies have made observations linking microglia to synapse removal in different disease models. In TBI, microglia appear to undergo a bimodal increase, with an early peak at 7 days followed by a later peak around 28 days (Simon *et al.*, 2017) (Fig. 1). Wake *et al.* (2009) used two-photon microscopy and found that transient cerebral ischaemia led to more prolonged contact between microglia and the presynaptic terminal, following which, several boutons were noted to have disappeared. Similarly, Hong *et al.* (2016) looked at a murine model of Alzheimer’s disease and observed early synapse (co-localized pre- and postsynaptic puncta) loss that preceded plaque formation. The authors described microglia engulfment of synaptic elements, which was not observed in mice with CR3 knockout (a high-affinity receptor expressed on macrophages) and a rescue of synaptic loss. They concluded that the complement cascade and microglia mediate early synaptic loss in Alzheimer’s disease. An *in vitro* study that aimed to model inflammation and ischaemia (two important pathologies in TBI) found evidence that the two processes act synergistically through microglial CR3 to induce long-term depression of synaptic transmission (Zhang *et al.*, 2014). These data point towards a potential role of microglia in synaptic pruning; however, there is a lack of conclusive mechanistic evidence, particularly in the context of TBI. It will therefore be of importance to address the role of microglia in the early and late phases of TBI.

Oligodendrocytes are the myelinating cells of the CNS, and are derived from the oligodendrocyte progenitor cell (OPC) lineage (Bradl and Lassmann, 2010). In a model of experimental demyelination, OPCs were observed to develop transient synaptic connections (evoked excitatory postsynaptic currents) with callosal axons prior to differentiation into oligodendrocytes during the remyelination phase (Etxeberria *et al.*, 2010). Myelin and axonal injury are well-described pathological processes in TBI (Armstrong *et al.*, 2016). A similar pattern of OPC and oligodendrocyte proliferation has been observed in the corpus callosum following an experimental model of TBI (Sullivan *et al.*, 2013). This may suggest that synapse-mediated axon-OPC interactions play a role in post-traumatic remyelination.

**Figure 3:**
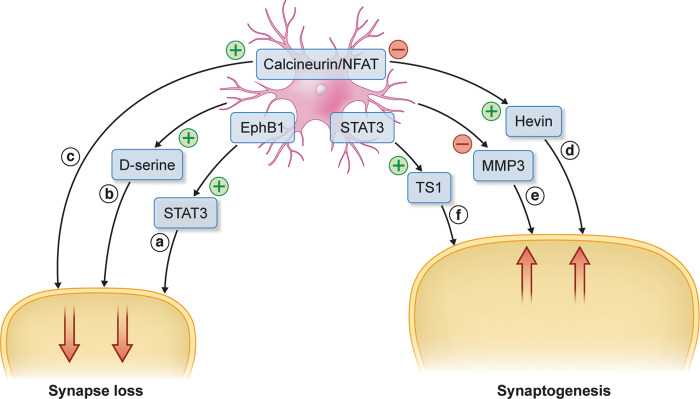
**Role of astrocytes in post-traumatic synapse loss and repair.** The upregulation of astrocytic ephrin B1 and STAT3 phosphorylation following TBI have been found to coincide with a decrease in VGlut1 (a) (Nikolakopoulou *et al.*, 2016). The tonic release of astrocytic d-serine has been associated with synapse damage after TBI (b) (Perez *et al.*, 2017). Astrocytic calcineurin/NFAT signalling pathway induces synaptic strength loss in CA1 and a reduction in synaptic protein loss (PSD95 and GluR1) (c) (Furman *et al.*, 2016). MMP3 has been implicated in maladaptive synaptic recovery after TBI (e) (Falo *et al.*, 2006). Hevin has been shown to promote post-traumatic synaptogenesis which was amplified by the blockade of the calcineurin/NFAT pathway (d) (Furman *et al.*, 2016). Thrombospondin 1 (which is regulated by STAT3) has been found to promote synapse recovery after motor neuron injury (f) (Tyzack *et al.*, 2014).

The role of the astrocyte has also been explored in the context of TBI. Furman *et al.* (2016) examined the effect of blocking the astrocytic calcineurin/nuclear factor of activated T cells (NFAT) signalling pathway on hippocampal synaptic function and protein levels. Using an adeno-associated virus delivery system to deliver VIVIT (an NFAT inhibitory peptide), the authors found a prevention of injury-induced synaptic strength loss in CA1 and an associated reduction in postsynaptic density protein loss (PSD95 and GluR1) (Fig. 3). Interestingly, these events occurred without a significant change in the number of astroglia between the treated and control rodents, indicating that the protective effects of VIVIT on the synapse do not require an associated reduction in glial activation. Astrocytes have also been implicated in propagating synaptic damage by the release of d-serine. Perez *et al.* (2017) found a shift from neuronal to astrocytic d-serine release (which activates NMDA receptors) after CCI. Using electrophysiology to measure synaptic transmission, the authors found that astrocyte-specific elimination of d-serine rescued synaptic plasticity (Fig. 3). Similarly, Nikolakopoulou *et al.* (2016) found evidence of the involvement of astrocytic ephrin B1 in synaptic remodelling after a moderate CCI in mice. After injury, an upregulation of ephrin B1 was observed that coincided with a decrease in the presynaptic protein VGlut1 (Fig. 3). Ablation of ephrin B1 accelerated the recovery of VGlut1 puncta. The authors linked ephrin B1 to STAT3 phosphorylation, which they implicated in synaptic remodelling. These studies suggest that astrocytes may play a role in synapse damage after TBI through a number of mechanisms.

To establish closer links between the inflammation response and the routes to synapse damage, it will be important to understand the complex intercellular interactions, their spatial relationships and the molecular mechanisms involved, which is being advanced by spatial transcriptomics and *in situ* sequencing methods (Chen *et al.*, 2020). These approaches combined with cell-type manipulation of molecular function (using genetic and optogenetic tools) could be used to dissect the temporal sequence of events at the molecular and cellular levels.

## The synapse, tau and progression of neurodegeneration after TBI

It has long been known that TBI can lead to neurodegeneration later in life. In 1928, the pathologist Harrison Martland published a seminal paper describing ‘punch drunk syndrome’, a constellation of chronic motor and neuropsychiatric symptoms in former boxers (Martland, 1928). The term dementia pugilistica was ascribed to the progressive neuropsychiatric condition that emerged after repetitive mild TBI, and a number of case series indicated a neuropathological basis to the syndrome (Corsellis, 1989; Roberts *et al.*, 1990). However, interest in the condition was limited up until the past decade when similar findings were made in non-boxing contact sports such as in American football and in military personnel (McKee *et al.*, 2009; Omalu *et al.*, 2011). The term CTE was introduced to describe the clinicopathological condition emerging from a wide range of physical exposures. One of the most consistent neuropathological findings in CTE is the presence of neurofibrillary tangles, which has led to it being described as a ‘tauopathy’ (McKee *et al.*, 2009). By contrast, the presence of amyloid-β (an important feature in Alzheimer’s disease) is a less consistent feature in CTE (Smith *et al.*, 2013*b*).

The synapse appears to play an important role in Alzheimer’s disease progression through its interaction with tau. In Alzheimer’s disease, tau systematically spreads through the brain, starting within the hippocampal formation then limbic and association cortices and finally to other parts of the brain (Braak and Braak, 1991). Trans-synaptic transmission has been suggested as the mechanism underpinning the circuit-driven spread of pathological tau in Alzheimer’s disease (De Calignon *et al.*, 2012; Liu *et al.*, 2012). In CTE, the anatomical progression of tau differs from that of Alzheimer’s disease, starting as scattered foci in the frontal lobe then progressing to the depth of sulci before spreading across the brain (McKee *et al.*, 2015). Interestingly, in CTE the medial temporal lobe is affected later in the disease, whereas in Alzheimer’s disease it is where the disease process starts. An experiment using P301S transgenic mice (which express mutant human tau) found accelerated spreading of phosphorylated tau after a single TBI, which the authors postulated to be synaptic-driven spread based on the progression pattern (Edwards *et al.*, 2020). Although this study could not make a conclusion on the mechanism underpinning the spread of tau, it does highlight this as an interesting area of study and the potential role that the synapse may play in the progression of neurodegeneration following TBI.

## The potential for synapse recovery after TBI

Several studies have identified the potential for synapse recovery after TBI, principally through the process of synaptogenesis (Fig. 1). Two studies examined reactive synaptogenesis using EM in preclinical models of TBI (Table 1). Scheff *et al.* (2005) found a partial recovery of hippocampal CA1 synapse (presynaptic terminal and postsynaptic density) density at 10 days post injury, which continued to Day 30 and reached up to 86% of control levels. Semchenko *et al.* (2006) used a rotatory head injury model in rats and found that an initial decrease in sensorimotor cortical synaptic (presynaptic terminal and postsynaptic density) density was followed by an increase from Day 7 to Day 30 post injury compared with control. Interestingly, a study by Canty using laser axotomy and two-photon microscopy observed regeneration in a subset of axons that then went on to form presynaptic boutons with prelesional densities (Canty *et al.*, 2013*a*). These observations point towards a degree of limited or maladaptive synaptic reorganization post injury.

The mechanisms underlying post-injury synaptogenesis have been investigated. Astrocytic matrix metalloproteinase 3 (MMP3), an enzyme involved with the breakdown of the extracellular matrix, has been implicated in maladaptive synaptic recovery after TBI (Huntley, 2012). A study comparing deafferentation alone with deafferentation and fluid percussion injury found persistently elevated MMP3 in both models, for which there was significant co-localization to astrocytes in the combined injury group (Falo *et al.*, 2006). Pharmacological inhibition of MMP3 led to increases in the thickening of the excitatory synapse postsynaptic density, measured using EM, and an improvement in spatial learning (Fig. 3). Similarly, Tyzack *et al.* (2014) examined the role of astrocytic STAT3 in synaptic (presynaptic terminal and postsynaptic density) remodelling in a facial murine nerve axotomy model. The authors implicated astrocytic STAT3-regulated release of thrombospondin-1 (THBS1) in post-injury synaptic remodelling (Fig. 3). The study found that knockout of the *Thbs1* gene led to impaired synaptic recovery, which mirrored findings in a model of cerebral ischaemia that showed deficits in synaptic density and functional recovery with knockout of *Thbs1*/*Thbs2* (Liauw *et al.*, 2008). In TBI, there is evidence of thrombospondin upregulation in both *in vitro* and *in vivo* models (Tran *et al.*, 2012; Wang *et al.*, 2016*b*). Plasma levels of THBS1 have also been found to be elevated in patients with TBI and may act as a prognostic biomarker (Wang *et al.*, 2016*b*). Astrocytes may also play a role in post-injury circuit reorganization by the secretion of Hevin, a synaptogenic protein (Singh *et al.*, 2016). Furman *et al.* (2016) found an increase in Hevin levels in injury mice compared with sham, which was further amplified by the blockade of the calcineurin/NFAT signalling pathway (Furman *et al.*, 2016) (Fig. 3). Together, current data suggest that astrocytes may play a double-edged role in synaptic reorganization post TBI, releasing enzymes and proteins that have been observed to both promote and impair synaptic recovery.

## Future perspectives

### Synapse diversity and selective synapse vulnerability

The importance of synapse diversity has not been considered in the context of TBI and is likely to be a crucial factor in understanding why particular synapses are damaged and others are resilient. Studies of the protein composition of individual synapses reveal that synapses are much more diverse than previously anticipated; each brain region has a particular signature of synapse composition, and the synapse proteomes of brain regions differ from one another (Micheva *et al.*, 2010; O’Rourke *et al.*, 2012; Broadhead *et al.*, 2016; Grant, 2018, 2019; Zhu *et al.*, 2018; Cizeron *et al.*, 2020). These differences encompass many proteins involved in disease processes, including neurodegeneration, excitotoxicity and inflammation (Roy *et al.*, 2018*a*, *b*). It is now possible to map the spatial distribution of molecular and morphological properties of individual synapses across the mouse brain and generate unbiased catalogues of synapse type, termed the ‘synaptome’ (the diversity of synapses in the brain or part thereof) (Zhu *et al.*, 2018; Cizeron *et al.*, 2020). Thus far, these brain-wide methods have been applied to excitatory postsynaptic markers (PSD95, SAP102). With a broader range of markers, including those in inhibitory and monoaminergic synapses and presynaptic terminals, these synaptomic approaches, combined with knowledge of the specific proteins and pathways involved with TBI, are likely to prove crucial in understanding TBI synaptopathy, providing powerful spatiotemporal insight into the vulnerable synapse types and brain regions. Synapse diversity and its spatial organization in the hippocampus have been shown to store behavioural representations, and alterations in the spatial organization in a mouse genetic model of schizophrenia produced altered representations (Zhu *et al.*, 2018). This suggests that TBI-induced changes in synapse diversity could underlie cognitive impairments. Synaptome mapping of the damaged synapses in TBI could also be used in conjunction with connectomic data to identify the affected circuits.

Synaptome mapping has been recently been applied at a brain-wide scale across the lifespan of the mouse revealing striking age-dependent differences in synapse composition (Cizeron *et al.*, 2020). The authors proposed that the different numbers of synapse types and subtypes at different ages would influence vulnerability to damage from inflammation, toxic proteins and brain injury. This further emphasizes the importance of understanding synapse diversity and selective vulnerability in TBI.

Synapses are also known to be dynamic at the level of protein composition and spine structure. *In vivo* imaging of dendritic spines in rodent brain shows that they are not stable structures, with a high percentage appearing to retract and rebuild each week (Lendvai *et al.*, 2000; Trachtenberg *et al.*, 2002; Holtmaat *et al.*, 2005). Furthermore, turnover studies indicate that the average half-life of the synapse proteome is a few days to weeks (Ehlers, 2003; Fornasiero *et al.*, 2018; Heo *et al.*, 2018), while synapse turnover rate appears to increase with ageing and in Alzheimer’s disease (Grillo *et al.*, 2013; Liebscher *et al.*, 2014). The ongoing restructuring of synapses raises the possibility that TBI could interfere with this process, perhaps preventing the production of certain types of synapses, and resulting in a net reduction in synapses. *In vivo* imaging of dendritic spines after TBI could be used to address the impact of TBI on the physical turnover of spines, and proteomic methods such as stable isotope labelling by amino acids in cell culture (SILAC) (Fornasiero *et al.*, 2018; Heo *et al.*, 2018) could be used to identify the synapse proteins affected. Ideally, simultaneous long-term imaging of pre- and postsynaptic terminals *in vivo* could reveal the temporal changes in synaptic architecture and their relationship to axonal and dendritic damage following TBI.

### Clinical monitoring of synapse damage using PET and fluid biomarkers

A range of experimental models have been used to examine the synapse following TBI. Of these, well-established rodent models such as the CCI and fluid percussion models are among the most used (Table 1). These models have strengths including their ability to replicate pathological sequelae (neuronal/axonal injury and inflammatory response) of TBI coupled to delivering a consistent and highly reproducible injury severity (Xiong *et al.*, 2013). However, they also have a number of important weaknesses such as the need for a craniotomy and the use of rodents whose lissencephalic brain structure limits their ability to duplicate the biomechanics of the human disease. Studies of human synapses in TBI have almost exclusively been restricted to post-mortem brain tissue, and there is a pressing need for methods enabling monitoring of synapse damage in living patients. The development of radiotracers that bind to the presynaptic protein synaptic vesicle glycoprotein 2A (SV2A) have shown promise as tools in PET imaging (Warnock *et al.*, 2014; Estrada *et al.*, 2016; Nabulsi *et al.*, 2016). The ^11^C-UCB-J ligand was found to be safe in first-in-human studies and had sufficient sensitivity to identify synapse loss in three patients with mesial temporal sclerosis (Finnema *et al.*, 2016).

Molecular imaging, although highly useful, requires a great deal of infrastructure and organization to implement, limiting its value as a diagnostic tool and for monitoring disease progression. Biomarkers from fluid samples that can be collected at the bedside would be very useful and pragmatic tools for use in the clinic. In recent years, there has been a growing body of research examining fluid biomarkers and looking at synaptic proteins in Alzheimer’s disease (Thorsell *et al.*, 2010; Brinkmalm *et al.*, 2014; Öhrfelt *et al.*, 2016; Galasko *et al.*, 2019; Lleó *et al.*, 2019). The CSF levels of a number of candidate proteins are significantly altered in patients with Alzheimer’s disease compared with control, including neurogranin, synaptosomal-associated protein 25 (SNAP-25), neuronal pentraxin 2 (NPTX2) and synaptotagmin (Thorsell *et al.*, 2010; Brinkmalm *et al.*, 2014; Öhrfelt *et al.*, 2016; Galasko *et al.*, 2019). Discovery proteomic approaches could be used to identify candidate synaptic proteins in TBI patients before taking these forward to clinical validation (Carlyle *et al.*, 2018). CSF is not routinely acquired as part of the TBI clinical management and obtaining a sample typically involves a lumbar puncture, which is invasive and carries risk. Blood biomarkers would be less invasive and easier to obtain. However, owing to the lower concentrations of neural proteins compared with CSF, blood sampling presents a challenge in identifying sensitive biomarkers.

### Synapse therapeutics

In Alzheimer’s disease, synapse loss correlates with early cognitive decline, making this pathological process an area of interest to target therapeutically (Scheff *et al.*, 2006; Jackson *et al.*, 2019). Although the relationship between synapse loss and cognitive impairment is less clear in TBI, there is some preclinical data suggesting that reduction in PSD95 is correlated with behavioural deficits in a murine TBI model (Wakade *et al.*, 2010). In models of Alzheimer’s disease, administration of neurotrophic factors such as ciliary neurotrophic factor (CNTF) and brain-derived neurotrophic factor (BDNF) has been found to rescue synapse loss and ameliorate cognitive decline (Nagahara *et al.*, 2009; Kazim *et al.*, 2014). An alternative approach is to target pathological processes that may drive synaptopathy. As detailed in this review, inflammation appears to play an important role in traumatic synaptopathy. Studies in other pathological processes including Alzheimer’s disease and schizophrenia have shown that amelioration of inflammation can reduce synapse loss (Hong *et al.*, 2016; Sellgren *et al.*, 2019). As the field of synapse therapeutics develops, a more nuanced approach that targets specific synapse types may be required. A study of Fragile X syndrome found that fenobam, an mGluR5 antagonist, impacted distinct synapse types, demonstrating that this therapeutic approach is feasible (Wang *et al.*, 2014). However, before synapse type-specific therapies are developed, the functional role of the various types needs to be better understood, as well as their role(s) in neurological disease.

## Conclusions

There have been important advances in our understanding of the role of the synapse in a range of brain disorders. The synapse appears to play an important role in TBI pathophysiology and is directly impacted by traumatic insult. TBI triggers multiple molecular and cellular mechanisms that interfere with synapse maintenance and potentially synapse production. To date, the approaches characterizing the temporal and spatial impact of TBI on the synapse have been limited, both in the scope of brain regions and the number of synaptic proteins examined. There is a need to utilize more systematic approaches that quantify large numbers of synapses across the whole brain, such as synaptome mapping (Zhu *et al.*, 2018). Coupled to this, the use of molecular imaging techniques is vital to translate preclinical findings of synaptic pathology after TBI into humans (Finnema *et al.*, 2016). A better characterization of traumatic synaptopathy, coupled to insights into the mechanisms that underpin it, is vital for the translation of effective therapies.

## Abbreviations

CCI =: controlled cortical impact
CR3 =: complement receptor 3
CTE =: chronic traumatic encephalopathy
EM =: electron microscopy
NFAT =: nuclear factor of activated T cells
PSD95 =: postsynaptic density protein 95
TBI =: traumatic brain injury
Wld^s^ =: Wallerian degeneration slow
